# Evaluation of intervertebral disc degeneration in young adult asymptomatic Dachshunds with magnetic resonance imaging and radiography

**DOI:** 10.1186/s13028-023-00702-0

**Published:** 2023-09-27

**Authors:** Vilma Liisa Jerosa Reunanen, Tarja Susanna Jokinen, Marjo Kristiina Hytönen, Jouni Juho Tapio Junnila, Anu Katriina Lappalainen

**Affiliations:** 1 Department of Equine and Small Animal Medicine, University of Helsinki, Helsinki, 00014 Finland; 2 Department of Medical and Clinical Genetics, University of Helsinki, Helsinki, 00014 Finland; 3 Folkhälsan Research Center, Helsinki, 00290 Finland; 4 Department of Veterinary Biosciences, University of Helsinki, Helsinki, 00014 Finland; 5 EstiMates Ltd, Turku, 20520 Finland

**Keywords:** Chondrodystrophic breed, *FGF4* retrogene, IVD, IVDD, MRI

## Abstract

**Background:**

Dachshunds have a high prevalence of intervertebral disc disease (IVDD) to which they are predisposed due to early intervertebral disc (IVD) degeneration and calcification. Moreover, the recently found 12-*FGF4* retrogene (RG) is associated with calcified discs visible on radiographs (CDVR) and IVDD. Earlier studies suggest that all IVDs of one-year-old Dachshunds show signs of degeneration. This prospective, analytical, blinded study aimed to investigate the extent and distribution of IVD degeneration in young adult (24–31 months) asymptomatic Dachshunds (n = 21) hypothesizing that not all IVDs of two-year-old Dachshunds are degenerated. Another aim was to explore the correlations between IVD degeneration evaluated with magnetic resonance imaging (MRI), the number of CDVR, and the dog’s 12-*FGF4*RG status. The study protocol included grading the CDVR on spinal radiographs, grading the IVD degeneration on T2-weighted sagittal and transverse high-field MR images of all IVDs (n = 546), and 12-*FGF4*RG variant genotyping.

**Results:**

Of all IVDs evaluated, 2% (n = 11) were normal based on MRI grading. Despite the study population having moderately degenerated IVDs (median MRI grade 3), there was also variation in the degree of IVD degeneration between individuals and in the distribution of IVD degeneration between different vertebral regions. The number of CDVR correlated significantly with the magnitude of IVD degeneration based on MRI evaluation and with the 12-*FGF4*RG genotype. The odds for being 12-*FGF4*RG homozygous were higher for Dachshunds with CDVR. However, the 12-*FGF4*RG variant did not alone explain the phenotypic variation in IVD degeneration.

**Conclusions:**

The number of CDVR is a valid indicator of overall IVD degeneration, as it correlates with MRI-based IVD grading. Also, as the extent and distribution of IVD degeneration varies between individual Dachshunds, selective breeding against IVDD using radiographic screening and 12-*FGF4*RG variant genotyping is possible.

## Background

Dachshunds have a high risk for intervertebral disc (IVD) degeneration and intervertebral disc disease (IVDD) at an early age [1–4]. The high IVDD prevalence of 15–30% in the breed makes it a major animal welfare problem causing pain, suffering, and increased mortality rate [3–6].

Pathogenesis of IVD degeneration in Dachshunds involves chondroid metaplasia, including replacement of notochordal cells within the nucleus pulposus by chondrocytes and fibrocartilage formation. In addition, nucleus pulposus loses proteoglycans and becomes dehydrated and finally potentially calcified [7–11]. Clinically, the degree of IVD degeneration and the number of calcified discs visible on radiographs (CDVR) affect the risk for IVDD [2, 4, 6, 11, 12]. Studies also show that many CDVR in the thoracolumbar area or presence of completely degenerated IVDs based on magnetic resonance imaging (MRI) clearly increase the risk of IVDD late recurrence after first episode with clinical signs of IVDD [13, 14].

The breed-typical disproportionate dwarfism, elicited by *FGF4* retrogene (RG) insertions in different genomic locations, is associated with premature IVD degeneration [15]. Dachshunds have two distinct *FGF4*RG insertions; one on chromosome 12 (12-*FGF4*RG, chondrodystrophy) and one on chromosome 18 (18-*FGF4*RG, chondrodysplasia) [16, 17]. All Dachshunds are homozygous for 18-*FGF4*RG [16] which defines their short-limbed morphology but does not seem to significantly affect the odds of CDVR [18]. However, 12-*FGF4*RG, which is nearly fixed in Dachshunds, is associated with CDVR and IVDD in multiple breeds [17, 18].

Mineralized IVDs are seen on radiographs, and this has been utilized in the screening programmes of Dachshunds, as the heritability of CDVR is high in the breed [19, 20]. Selective breeding utilizing radiographic screening at the age of 24–48 months is a proven method for reducing IVDD risk in Dachshunds [2, 4, 6, 21].

Unlike radiographs, MRI allows detection of non-mineralized stages of disc degeneration [22, 23]. It is the most accurate imaging modality for evaluating the degree of IVD degeneration, and MRI grading of IVD degeneration correlates with the degree of histologically visible IVD degeneration [23–25]. Pfirrmann grading system using T2-weighted MRI is well established for evaluating IVD degeneration in humans. It is based on MRI signal intensity, disc structure, distinction between nucleus pulposus and anulus fibrosus, and disc height [26]. Furthermore, it has been proven suitable for grading canine IVD degeneration using T2-weighted sagittal plane low-field MRI [27] and more recently high-field transverse plane MRI [28].

For the last decades, since Hansen’s studies on canine IVD degeneration pathology [7], the general belief in veterinary medicine has been that all IVDs in Dachshunds degenerate at a young age. However, no prospective MRI studies classifying IVDs of young Dachshunds exist. The main aim of this study was to investigate the extent and distribution of IVD degeneration in young Dachshunds with high-field MRI. This was accomplished through modified Pfirrmann grading system combining sagittal and transverse MR images of IVDs. A second aim was to explore the correlations between the number of CDVR, the IVD degeneration evaluated with MRI, and the dog’s 12-*FGF4*RG status. We hypothesized that not all IVDs of two-year-old Dachshunds are degenerated, the degree of IVD degeneration varies between vertebral regions and that the degeneration status in MRI correlates with the number of CDVR and the 12-*FGF4*RG status.

## Methods

### Animals

Client-owned Dachshunds aged 24–31 months were recruited to this prospective, analytical, blinded study. We aimed to increase the possibility of finding Dachshunds with relatively less degenerated IVDs and consequently more 12-*FGF4*RG genotypic variation. Thus, we used Finnish Kennel Club’s routine radiographic IVDD screening results as inclusion criteria. We included dogs with either their own IVDD screening result with a maximum of two CDVR or their parents’ screening results with a maximum of two CDVR. Exclusion criteria were any signs of systemic or neurologic illness.

The study protocol was approved by the Project Authorization Board of the Regional State Administrative Agency of Southern Finland (ESAVI/ 29986/ 2020). Participation was voluntary and all examinations were performed between January 2021 and January 2022 at the Veterinary Teaching Hospital, University of Helsinki, with the owners’ informed and written consent. Recruitment was performed by social media advertisement during the year 2021.

### Clinical evaluation of dogs

To ensure absence of clinical signs, a patient history was obtained from the owner and a physical examination was performed by one of the authors (VLJR). Next, a neurological examination was performed by a board-certified veterinary neurologist (TSJ). This included evaluation of mental status, posture, gait, postural reactions, spinal reflexes, cranial nerves, and possible spinal pain on palpation.

### Anaesthesia and diagnostic imaging

Radiographs were taken and MRI was conducted under general anaesthesia for all dogs. Haematocrit and serum protein and creatinine concentration were analysed before anaesthesia. The anaesthesia protocol was planned individually by the presiding hospital anaesthesiologist. Dogs were discharged after full recovery from general anaesthesia.

Spinal radiographs were taken with indirect (CPI Indico 100 RAD 150 kV 2006, Fujifilm FCR XG-1) or direct (Arcoma Intuition DR 125 kV 630 mA, Canon CXDI-401 C) digital imaging technique. The Finnish Kennel Club spinal imaging protocol was utilized; thus, the set consisted of laterolateral radiographs of the cervical spine, cervicothoracic junction, thoracic spine, thoracolumbar junction, and lumbar spine including sacrum [29]. This same protocol is utilized internationally and has been described previously [19, 21, 30].

All MRI examinations were conducted in dorsal recumbency with a 1.5 Tesla scanner (Phillips, Ingenia 1.5T S, Philips Medical System) using head and spine coils. The MRI protocol was a combination of two previously reported protocols for imaging and grading IVD degeneration [27, 28]. Turbo-Spin-Echo T2-weighted sagittal and transverse plane MRI was performed for the cervical, thoracic, and lumbar spine. Imaging parameters (TR for sagittal imaging 3020 ms, TR for transverse imaging 3648 ms, TE 100 ms, flip angle 90°, slice thickness 2.5 mm) were chosen according to the requirements for evaluation of IVDs.

### Image analyses and genetic analyses

Blinded radiographic evaluation of IVDs was performed and recorded in a random order on the sets of radiographs by an experienced radiologist (AKL). Every IVD space was graded as calcified or not [30, 31]. Briefly, a normal, totally radiolucent IVD with a normal width of the IVD space was graded as zero (0 = normal). An IVD with a mineralization, regardless of the size and opacity of the mineralization was graded as one (1 = calcified) [4, 19]. Thereafter, the number of CDVR was calculated for every dog and for each IVD space.

**Fig. 1:**
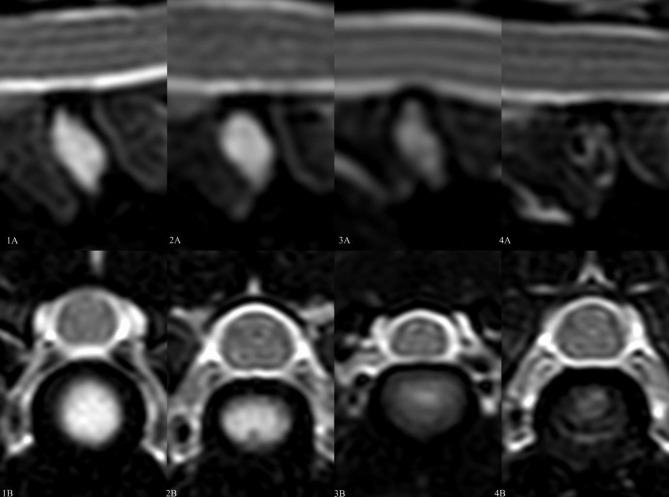
Model images of each MRI grade in the two image plane classification scheme of IVD degeneration. MRI = magnetic resonance imaging; IVD = intervertebral disc. 1 A sagittal and 1B: transverse plane image of non-degenerated MRI grade 1 intervertebral disc (IVD). 2 A sagittal and 2B: transverse plane image of mildly degenerated MRI grade 2 IVD. 3 A sagittal and 3B: transverse plane image of moderately degenerated MRI grade 3 IVD. 4 A sagittal and 4B: transverse plane image of severely degenerated MRI grade 4 IVD.

For MRI grading of the IVDs, the authors (TSJ and VLJR) developed a combined two image plane classification scheme previously validated separately for transverse and sagittal images [26–28] (Table 1), including model images of each grade (Fig. 1). The purpose of the two-image plane classification was to increase the authors subjective certainty in IVD grading.

**Table 1:** Two image plane MRI classification scheme for IVD degeneration

MRI grade	IVD structure	Distinction of nucleus and anulus	IVD signal intensity	IVD Width	Grade of IVD degeneration
1	Homogeneous, bright white. Vertical band is accepted in sagittal plane if transverse plane is homogeneous and bright white.	Clear	Hyperintense or isointense to cerebrospinal fluid	Normal	Normal / healthy IVD
2	Inhomogeneous with or without vertical bands. Maximum one-third of the nucleus pulposus is hypointense.	Clear	Hyperintense or isointense to cerebrospinal fluid	Normal	Mild degeneration
3	Inhomogeneous, grey. Minimal black spot is accepted if it is only in one image plane.	Unclear, but visible at least in one image plane	Intermediate: isointense or hypointense to cerebrospinal fluid	Normal to slightly decreased	Moderate degeneration
4	Inhomogeneous, grey to black.	Lost	Intermediate or hypointense to cerebrospinal fluid	Normal to moderately decreased	Severe degeneration
5	Inhomogeneous, black.	Lost	Hypointense to cerebrospinal fluid	Collapsed IVD space	Severe degeneration and IVD herniation

MRI, magnetic resonance imaging; IVD, intervertebral disc

Modified according to previous literature [26–28]

Before the actual blinded classification of the anonymized MR images, the two authors evaluated together seven non-anonymized MR image sets (182 discs) to practise and synchronize the grading. Thereafter, the evaluators, blinded to the signalment and radiographic results, independently graded every IVD of anonymized sets of MR images in random order. Immediately afterwards, the grade for each disc was determined by the consensus of the two evaluators. The MRI grades of all 26 IVDs were summed for each dog. This parameter (MRI total sum) describes the degree of overall IVD degeneration of an individual dog. The minimum MRI total sum, 26, is achieved when all IVDs are normal (MRI grade 1) and the maximum would be 130, if all IVDs were severely degenerated and herniated (MRI grade 5).

Three millilitres of whole blood sample (EDTA) were collected from each dog while they were sedated. The blood samples were stored at -18 °C. After all samples were collected, they were sent for 12-*FGF4*RG and 18-*FGF4*RG variant genotyping to a commercial laboratory (LABOKLIN GmbH & Co. KG, Bad Kissingen, Germany).

### Statistical methods

To investigate the association between the overall IVD degeneration and the number of CDVR, a linear regression model was fitted for the association between the number of CDVR and the MRI total sum, having the MRI total sum as the response variable. In addition, as the IVD calcification indicates a more advanced stage of IVD degeneration [7], we investigated the association between the severely degenerated MRI grade 4 IVDs and the number of CDVR. It was analysed utilizing Poisson regression models, having the number of MRI grade 4 IVDs as the response variable.

The associations between the 12-*FGF4*RG variant and the presence of CDVR (with or without CDVR), MRI total sum, and the number of MRI grade 4 IVDs were each assessed with logistic regression applying Firth’s penalized likelihood. Odds ratios (OR) with 95% confidence interval (CI) were calculated from the logistic regression models.

To investigate the distribution of IVD generation within different vertebral regions, the proportions of normal or only mildly degenerated MRI grade 1–2 IVDs were compared between the three vertebral regions (cervical, thoracic, lumbar) using a logistic regression model.

The effects of demographic variables (age, sex, weight) on the number of CDVR were assessed simultaneously with a Poisson regression model, and the effects of the demographic variables on the MRI total sum were evaluated with a multivariate ANOVA model.

All statistical analyses were performed by a professional statistician (JJ) using SAS System for Windows, version 9.4 (SAS Institute Inc., NC USA). P-values < 0.05 were considered significant.

## Results

Altogether 21 clinically normal Dachshunds (546 IVDs) were included (seven Standard Smooth-haired, five Standard Long-haired, four Standard Wire-haired, three Miniature Long-haired, one Miniature Wire-haired, and one Miniature Smooth-haired). Their mean age was 27 months (SD 2.4 months, range 24–31 months) and mean weight 7.9 kg (SD 1.8 kg, range 4.2–10.9 kg). Of the Dachshunds, 12 were females and nine males (two neutered).

All dogs had normal physical and neurological examinations. Eight dogs were eligible according to their own previous CDVR grading result and ten dogs according to parents’ CDVR grading results. Additionally, three Dachshunds with no previous CDVR grading results (parents’ or own) were included in the study.

**Fig. 2:**
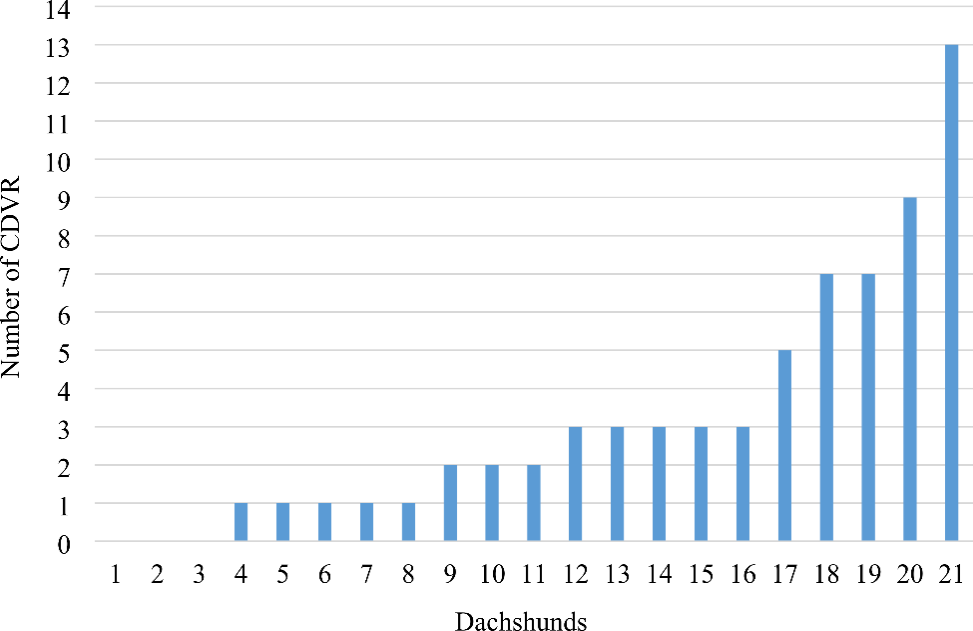
Distribution of calcified discs visible on radiographs (CDVR) per dog. CDVR = calcified discs visible on radiographs

The CDVR distribution is presented in Fig. 2. The median number of CDVR per dog was two (range 0–13). Of all 546 IVD spaces, CDVR were observed in 67 (12%). Most often CDVR were detected in IVD spaces C7-T1 and T5-T6 (both in five dogs), whereas no CDVR were observed in L6-L7 and L7-S1.

On MRI evaluation, six dogs (29%) had MRI grade 1 IVDs (1–4 per dog), 12 dogs (57%) MRI grade 2 IVDs (1–6 per dog), 21 dogs (100%) MRI grade 3 IVDs (12–26 per dog), and 18 dogs (86%) MRI grade 4 IVDs (1–13 per dog). None of the dogs had MRI grade 5 IVDs. The median MRI grade for all examined IVDs was 3.

**Fig. 3:**
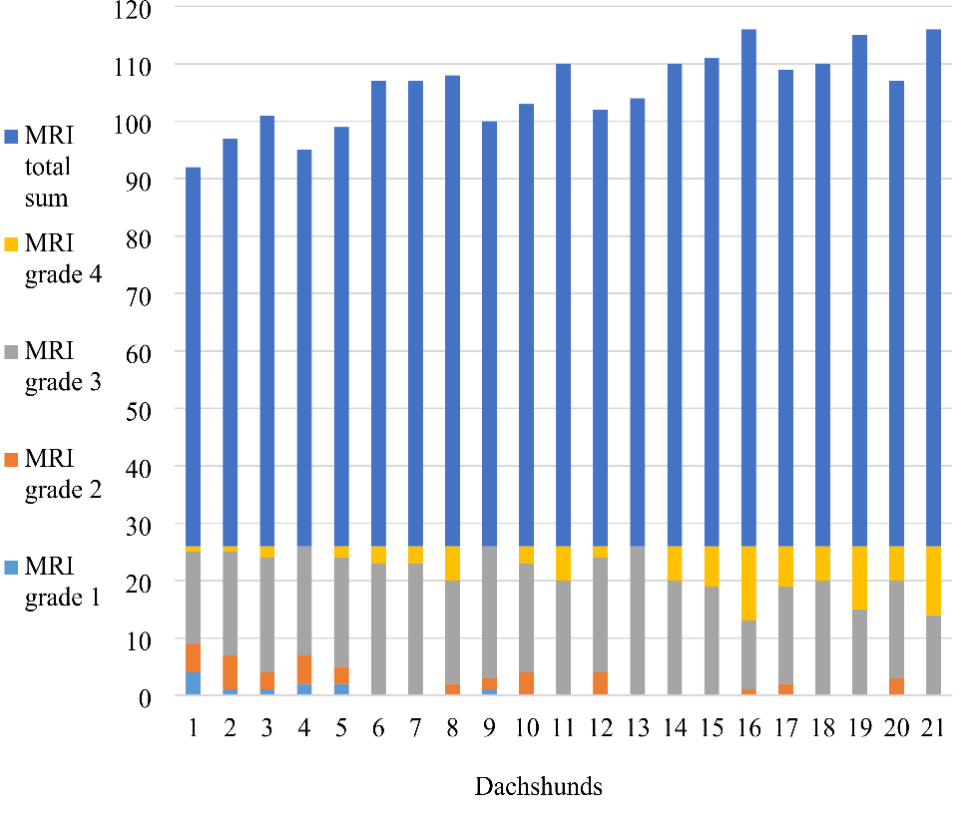
Distribution of different MRI grades (1–4) and overall IVD degeneration per dog. MRI = magnetic resonance imaging; IVD = intervertebral disc; MRI grade 1 = non-degenerated IVD; MRI grade 2 = mildly degenerated IVD; MRI grade 3 = moderately degenerated IVD; MRI grade 4 = severely degenerated IVD. X-axis represents the 21 Dachshunds. The MRI grading results of each dog are at the bottom of the Y-axis and the MRI total sum is at top of the Y-axis. The MRI total sum indicates the degree of overall IVD degeneration in each individual and is calculated by summing MRI grades of all 26 discs. The higher the column, the more advanced the overall IVD degeneration

**Fig. 4:**
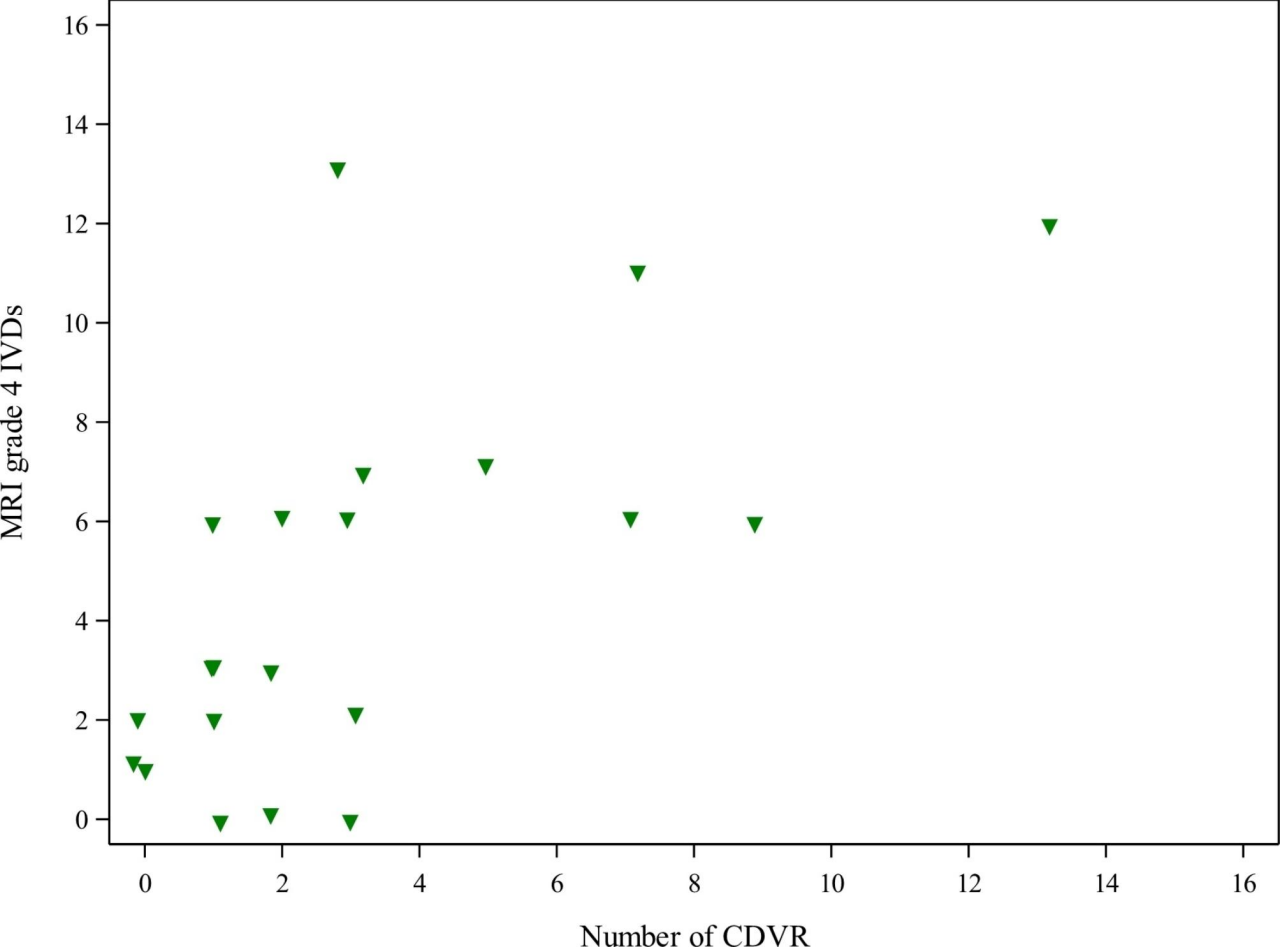
Correlation between the number of calcified discs visible on radiographs and MRI grade 4 discs. MRI = magnetic resonance imaging; IVD = intervertebral disc; MRI grade 4 = severely degenerated IVD. Scatter plot visualizing the correlation between the number of calcified discs visible on radiographs (CDVR) and the number of IVDs with MRI grade 4 degeneration. A strong association emerged between the number of CDVR and the number of MRI grade 4 discs based on Poisson regression analysis (estimate 1.13, 95% CI 1.08–1.19, P < 0.0001)

The median MRI total sum, calculated for each dog separately, was 81 (range 66–90) (Fig. 3). A strong association emerged between the number of CDVR and the MRI total sum and between the number of CDVR and the number of MRI grade 4 IVDs. An increasing number of CDVR resulted in higher MRI total sum (P = 0.002, linear regression estimate 1.28, CI 0.52–2.03) and increased number of MRI grade 4 IVDs (P < 0.0001, Poisson regression estimate 1.13, CI 1.08–1.19) (Fig. 4).

**Fig. 5:**
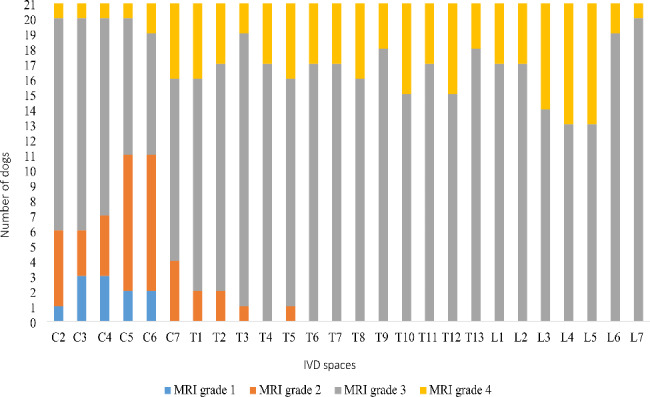
Distribution of MRI grades between intervertebral disc (IVD) spaces. MRI = magnetic resonance imaging; IVD = intervertebral disc; MRI grade 1 = non-degenerated IVD; MRI grade 2 = mildly degenerated IVD; MRI grade 3 = moderately degenerated IVD; MRI grade 4 = severely degenerated IVD.

In all 546 IVDs of examined dogs, the distribution of MRI grades was as follows: 11 MRI grade 1 (2%), 40 MRI grade 2 (7%), 395 MRI grade 3 (72%), and 100 MRI grade 4 (18%) (Fig. 5). A significant difference in IVD degeneration existed between vertebral regions. The probability for IVDs to be graded as normal or mildly degenerated (MRI grade 1 or 2) was significantly higher in the cervical region than in the thoracic or lumbar region (P < 0.0001 and P = 0.0004), but no significant difference emerged between thoracic and lumbar regions (Table 2).

**Table 2:** The probability of MRI grades 1 and 2 versus MRI grades 3 and 4 IVDs according to the vertebral region

Vertebral region	MRI grade 1 + 2, n (%)	MRI grade 3 + 4 n (%)	Comparison between vertebral regions	*Odds ratio	95% CI	P-value
Cervical spine	45 (36%)	81 (64%)	Lumbar versus cervical spine	0.006	0.0004–0.10	0.0004
Thoracic spine	6 (2%)	267 (98%)	Thoracic versus cervical spine	0.04	0.018–0.10	< 0.0001
Lumbar spine	0 (0%)	147 (100%)	Thoracic versus lumbar spine	7.17	0.397–129.36	0.18

MRI, magnetic resonance imaging; IVD; intervertebral disc; MRI grade 1 = non-degenerated IVD; MRI grade 2 = mildly degenerated IVD; MRI grade 3 = moderately degenerated IVD; MRI grade 4 = severely degenerated IVD; n = number of intervertebral discs

*Logistic regression Odds ratios for the probability of MRI grades 1 and 2; 95% CI, 95% confidence interval

The demographic variables (age, sex, or weight) were not significantly associated with the MRI total sum (P = 0.32, P = 0.58, and P = 0.25) or the number of MRI grade 4 discs (P = 0.55, P = 0.73, and P = 0.46). Neither were sex nor weight significantly associated with the number of CDVR (P = 0.77 and P = 0.69). Moreover, when one extreme outlier, the youngest dog with 13 CDVR (highest of the range) was removed from the analysis, age ceased to show a significant association with the number of CDVR (P = 0.17).

Three Dachshunds were heterozygous and 18 were homozygous for the 12-*FGF4*RG variant. The heterozygous dogs were all standard-size Dachshunds and the only ones without CDVR. In addition, they represented all coat types (wire-, smooth-, and long-haired). The probability of being homozygous for the 12-*FGF4*RG variant was clearly higher for dogs with CDVR than for dogs without CDVR but with a broad confidence interval, due to the low number of observations (OR 259, CI 2.9-22802). As expected, all Dachshunds were homozygous for the 18-*FGF4*RG variant.

The median MRI total sum, indicating the degree of the dog’s overall IVD degeneration, was lower (71, range 66–75) for 12-*FGF4*RG heterozygous dogs than for homozygous dogs (82, range 69–90). Still, a statistical association between MRI total sum and the 12-*FGF4*RG variant could not be shown with the current data (OR 1.30, CI 0.99–1.71 for MRI total sum).

## Discussion

We investigated the extent and distribution of IVD degeneration in clinically healthy young adult Dachshunds with high-field MRI in this prospective, analytical, blinded study. To our knowledge, this is the first prospective study using high-field MRI for canine IVD degeneration. We observed variation in IVD degeneration including some non-degenerated IVDs in our study population.

Our results contradict the generally accepted conclusion in veterinary medicine that all IVDs of young adult Dachshunds are degenerated, as six dogs had altogether 11 non-degenerated IVDs based on MRI evaluation. Since IVD degeneration and the number of CDVR are highly heritable [6, 17–20], we expected to gain a study population of Dachshunds with relatively less degenerated IVDs and more phenotypic and genetic variation by including dogs according their own or parents’ radiographic results. Still, only 2% of all IVDs were non-degenerated and our MRI grade median of 3 was consistent with moderate overall IVD degeneration. In contrast, Kranenburg et al. [25] reported a Pfirrmann grade median of 4 (severe IVD degeneration) for older chondrodystrophic dogs with symptomatic IVDD and a median of 3 for older non-chondrodystrophic dogs with symptomatic IVDD. The discrepancy is explained with our inclusion criteria. However, it is notable that the IVD degeneration of these young clinically healthy Dachshunds is equivalent to older non-chondrodystrophic dogs with IVDD, consequently suggesting a similar risk for IVDD in these two groups.

We observed that some Dachshunds in our study had variation in IVD degeneration and some did not. In other words, individual dogs had 0–15% of non-degenerated IVDs and all MRI grades were found in some individuals, but some had only MRI grade 3 and 4 IVDs. This variation between young asymptomatic adult Dachshunds has not been investigated before with MRI grading. Moreover, it is an important finding because without variation selective breeding against IVDD would be impossible.

Our study shows, based on MRI grading, an interesting and statistically significant difference in IVD degeneration between the vertebral regions. We observed less IVD degeneration in the cervical spine than in the thoracic and lumbar spine. These findings are parallel with the role of premature IVD degeneration in IVDD of chondrodystrophic breeds, as the localization of their IVDD is most often in the thoracolumbar region [7, 12, 14, 32]. The current study is the first prospective study utilizing MRI-based grading of IVD degeneration for young adult dogs. The uneven distribution of IVD degeneration in our data differs from the study of Hansen [7], which had suggested that early IVD degeneration occurs simultaneously in all intervertebral spaces of chondrodystrophic dogs.

Of all 21 Dachshunds, three did not have any CDVR. Furthermore, when calculating the MRI total sum for each dog to describe their overall IVD degeneration, we noticed that the median of MRI total sums of these three dogs were about ten units below the overall median. In addition, a strong statistical association existed between the number of CDVR and the number of severely degenerated (MRI grade 4) discs and between the number of CDVR and the MRI total sum, indicating dogs’ overall IVD degeneration. Our findings suggest that in young adult Dachshunds the number of CDVR reflects the frequency of severely degenerated IVDs and that the radiographic grading and the number of CDVR are reliable indicators of overall IVD degeneration. Similar conclusions have been reported earlier about the association between CDVR and clinical IVDD [2, 4, 6, 11]. IVD degeneration appears to be somewhat higher based on MRI grading than based on CDVR which is in accordance with previous reports [12, 33]. Computed tomography has also been shown to be a more effective imaging method than radiography in detecting calcified IVDs [11, 34].

Our study population included three 12-*FGF4*RG heterozygous dogs, which allowed us to investigate the possible correlation of the number of alleles (heterozygous versus homozygous) with IVD degeneration. These heterozygous individuals were also the only ones without CDVR and with lower overall degeneration level based on MRI total sum. We detected a statistically significant association between the 12-*FGF4*RG allele number and the dog’s CDVR status, which somewhat contradicts an earlier report [6]. They found a perfect sensitivity (1.0) but very low specificity (0.14) in wire-haired Dachshunds and no association in smooth- or long-haired Dachshunds. However, the setup was different, as they compared dogs with less than five CDVR and dogs with more than five CDVR, while we compared dogs with or without CDVR. This different setup can explain the partial discrepancy between the two studies.

We detected variation in IVD degeneration between young adult asymptomatic Dachshunds, which cannot completely be explained by homozygosity or heterozygosity of the 12-*FGF4*RG variant. Three 12-*FGF4*RG homozygous Dachshunds had MRI grade 1 IVDs and lower MRI total sum than one of the 12-*FGF4*RG heterozygous Dachshunds. The findings cannot be explained by demographic variables, as age, sex, or weight did not have a significant association with MRI total sum. Moreover, this variation cannot be explained by the 18-*FGF4*RG variant either, as all Dachshunds in our study were homozygous for this variant. Our results reinforce the previous conclusion of Bruun et al. [6] that the two currently recognized *FGF4*RG variants do not explain all phenotypic variation in IVD degeneration and IVDD in Dachshunds. In addition, other complicated genetic or environmental factors might exist [18, 35]. Also earlier studies state that IVDD is a multifactorial disease [2, 15, 36].

We used high-field T2-weighted MRI and a combined two image plane MRI grading to evaluate IVD degeneration as accurately as possible. When evaluating IVDs of small dogs, MRI resolution may hinder the evaluation and the difference between two Pfirrmann grades might not be clear in every IVD [26, 27]. With two image plane evaluation, our purpose was to achieve higher subjective certainty when detecting discriminating features as homogeneous versus inhomogeneous and differentiating nucleus pulposus and anulus fibrosus in the group of dogs where the smallest one weighted only 4.9 kg.

The main limitation in this study is the small sample size of only 21 Dachshunds when the 12-*FGF4*RG allele frequency is extremely high in the breed. Investigating the correlation between 12-*FGF4*RG and IVD degeneration was not the main objective of this study. However, we achieved a statistically significant result for the association between the 12-*FGF4*RG variant and the presence or absence of CDVR, but the precision was quite low. Moreover, the statistical association of the MRI total sum with the 12-*FGF4*RG variant remains unclear due to our limited data, warranting future studies with larger study populations. An additional limitation in our study is the absence of intra- or interrater analysis for our IVD grading based on two plane MRI evaluation. However, these methods have previously been separately validated for grading canine IVD degeneration [26–28]. As we did not specifically address this issue in our study, further studies of sensitivity and specificity of combined two-image plane grading are warranted.

## Conclusions

This study confirms that although IVD degeneration of young adult Dachshunds without neurological signs is substantial these dogs also have non-degenerated IVDs. Moreover, there is variation in the extent of IVD degeneration between individual Dachshunds and in the distribution of IVD degeneration between different vertebral regions. The number of CDVR can be used as an indicator for magnitude of MRI-graded IVD degeneration. Despite the correlation with the number of CDVR, the 12-*FGF4*RG variant alone does not explain the phenotypic variation in IVD degeneration in Dachshunds. IVDD is a significant animal welfare issue, especially for many chondrodystrophic breeds. Therefore, for effective breeding against premature IVD degeneration and IVDD, the full potential of radiographic screening and genetic testing should be used.

## Data Availability

The data will not be made openly available to third parties or outside the original research team for patient confidentiality reasons.

## Declarations

### Prior publication

Data have not been published previously.

## Abbreviations

CDVR: Calcified discs visible on radiographs
IVD: intervertebral disc
IVDD: intervertebral disc disease
MRI: Magnetic resonance imaging
12-*FGF4*RG: *FGF4* retrogene insertion on chromosome 12, chondrodystrophy
18-*FGF4*RG: *FGF4* retrogene insertion on chromosome 18, chondrodysplasia
